# Editorial: Neural Tracking: Closing the Gap Between Neurophysiology and Translational Medicine

**DOI:** 10.3389/fnins.2022.872600

**Published:** 2022-03-16

**Authors:** Giovanni M. Di Liberto, Jens Hjortkjær, Nima Mesgarani

**Affiliations:** ^1^School of Computer Science and Statistics, Trinity College Dublin, Dublin, Ireland; ^2^ADAPT Centre, d-real, Trinity College Institute for Neuroscience, Dublin, Ireland; ^3^Hearing Systems Group, Department of Health Technology, Technical University of Denmark, Kongens Lyngby, Ireland; ^4^Electrical Engineering Department, Zuckerman Mind Brain Behavior Institute, Columbia University, New York, NY, United States

**Keywords:** speech perception, neural entrainment, EEG, MEG, music perception, neuromarker, hearing impairment, aging

Perception involves making sense of the world around us by processing a continuous flow of multi-modal sensory information. In doing so, the human brain produces electrical activity that can be measured in a variety of scenarios and tasks to shed light on the neural basis of continuous perception. This work has shown that electrical brain activity synchronizes to particular properties of sensory inputs, a phenomenon referred to as *neural tracking* (Obleser and Kayser, 2019). Recent work demonstrated that both invasive and non-invasive electrophysiology recordings can robustly detect neural tracking (Lalor et al., 2006; Ding and Simon, 2012; Gross et al., 2013; Zion Golumbic et al., 2013), offering objective measurements to study perception in increasingly more complex tasks involving continuous real-life stimuli, such as speech and music.

The case of auditory perception is particularly remarkable. The discovery that neural signals reliably track the amplitude envelope of continuous sounds (*envelope tracking*) (Lalor et al., 2009) has led to new research directions. *In primis*, envelope tracking measurements have enabled a range of studies on auditory attention in realistic multi-talker scenarios (e.g., see COCOHA project, H2020.2.1.1.4. ID = 644732), showing that signals recorded with invasive electrocorticography (ECoG) as well as non-invasive electro- and magneto-encephalography (EEG/MEG) track attended and unattended sounds in a different manner (Ding and Simon, 2012; Zion Golumbic et al., 2013; O'Sullivan et al., 2014, 2019). This pioneering discovery led to an entire new direction for brain-computer interface research, with perspectives for novel devices such as brain-controlled hearing-aids (Eyndhoven et al., 2017; O'Sullivan et al., 2017; Ceolini et al., 2020). A parallel line of work demonstrated that multiple properties of the same stimulus are tracked simultaneously (O'Sullivan et al., 2016; Di Liberto et al., 2021a; Gillis et al., 2021). In the context of speech listening, cortical signals were shown to track progressively higher-level properties of the speech signal, from acoustical features (Lalor and Foxe, 2010; Ding et al., 2014) to linguistic units (Di Liberto et al., 2015, 2018b; Brodbeck et al., 2018; Lesenfants et al., 2019), prosody (Myers et al., 2019; Teoh et al., 2019), and semantic content (Broderick et al., 2018, 2021; Weissbart et al., 2020). As such, neural tracking measurements can offer a rich view into the hierarchical encoding of speech by providing us with distinct objective indices referring to different processing stages.

The outstanding advances in this domain have pushed scientists to explore the potentialities of studying neural tracking in translational research (Jessen et al., 2019; Dial et al., 2021; Geirnaert et al., 2021; Palana et al., 2022). Indeed, the unprecedented opportunity to assess the speech processing hierarchy as a whole (as well as for other stimuli, such as music) in a single experimental session is a very compelling reason that encourages the exploration of translational research directions. Furthermore, the possibility of using ecologically-valid tasks, such as movie or cartoon watching, opens the door to cohorts that would be difficult to assess otherwise (Di Liberto et al., 2018a; Jessen et al., 2019; Attaheri et al., 2022). Nevertheless, the feasibility for the translational applications of neural tracking metrics remains to be determined, as the theoretical and methodological challenges are yet to be uncovered.

In this special topic issue we have gathered contributions from scientists working in diverse disciplines who have common interests in the neural tracking phenomenon from various research domains. The current issue includes studies on speech (Alickovic, Ng, et al.) and music perception (Hausfeld et al.), selective attention (Huet et al.), and aging in healthy individuals (Mesik et al.). It also covers methodological considerations for translational research (Crosse et al.) and for measuring responses to different speech features (Bachmann et al.), as well as theoretical and practical perspectives on hearing-impairment (Alickovic, Ng, et al.), hearing-aid technology (Alickovic, Lunner, et al.), and schizophrenia (Meyer et al.). Bringing together work from a variety of research domains demonstrates the extensive width of applications for neural tracking research, while hopefully helping to build a new community of interdisciplinary research. We were very fortunate to enlist a varied and talented group of authors to contribute such a wide range of topics. Thirty-five authors contributed to the eight papers included, with a mixture of six original research articles, one review, and one hypothesis and theory. Taken together these papers present an overview of research on neural tracking from a range of perspectives, indicating a promising research framework that can greatly contribute to translational research questions, both from theoretical and applied perspectives.

As typical for new lines of work, the literature offers a diverse set of approaches and views regarding neural tracking. One issue is the apparent inconsistency in the terminology used by different research groups, leading to some confusion with terms such as neural entrainment, synchronization, and tracking. Obleser and Kayser (2019) have recently put forward an important distinction between the concepts of neural entrainment in the *narrow* and *broad sense*. In their view, neural entrainment in the *narrow sense* refers to the concept of “synchronization,” whereby endogenous self-sustained neural oscillators adjust their temporal dynamics (“rhythms”) to that of the sensory input (Schroeder and Lakatos, 2009). While this definition is specific to a particular neural mechanism, we use the term *neural tracking* to refer to neural entrainment in the broad sense, where the neurophysiology measurements likely reflect a combination of multiple phenomena. In fact, it is challenging (to say the least) to make any claim on the specific neural mechanisms generating such non-invasively recorded signals. Nevertheless, a somewhat agnostic view on such underlying neural mechanisms would not prevent us from making valuable theoretical and practical use of such measurements. Work using such measures has already contributed to our understanding of speech (Mesgarani et al., 2014; Di Liberto et al., 2015, 2021a; Ding et al., 2015; Brodbeck et al., 2018; Broderick et al., 2018) and music perception (Tal et al., 2017; Di Liberto et al., 2020, 2021b; Marion et al., 2021; Zuk et al., 2021), selective attention (O'Sullivan et al., 2014; Decruy et al., 2020; Fuglsang et al., 2020), multisensory integration (Crosse et al., 2016; Sullivan et al., 2021), and even abstract cognitive processes such as arithmetic (Kulasingham et al., 2021). The work in this Research Topic attempts to portray a wide set of findings while using consistent terminology.

This Research Topic is a first attempt to put together methodological, theoretical, and applied work with the common aim of projecting the study of neural tracking toward translational research. Recent reviews have discussed the neural tracking phenomenon (Obleser and Kayser, 2019; Hamilton and Huth, 2020), including specific applied research scenarios involving atypical cohorts (Palana et al., 2022). From that work, it is clear that we have only scraped the surface of a line of work with great potential, and that much more is yet to come. Neural tracking has a minimal presence in translational research at present. One challenge is that the literature portrays a complex research landscape, including many methodologies to evaluate and report the results. As for more established methodologies (e.g., ERPs), the definition of appropriate standardisations and the development of appropriate tools to more rapidly and effortlessly measure neural tracking are crucial to effectively adopting these methodologies to translational research.

One paper in this article collection contributed to this debate, presenting a set of precise guidelines on how to measure, evaluate, and report neural tracking in applied research by using one particular approach (the multivariate temporal response function—mTRF) (Crosse et al.). Others have emerged from discussions at conferences (e.g., ARO) and workshops (e.g., the Telluride Neuromorphic Engineering workshop), with special sessions revolving around neural tracking. The more specific *Cognition and Natural Sensory Processing* (CNSP) initiative, which has an educational focus, aims at bringing together researchers interested in studying and using neural tracking measurements, offering a workshop and online resources, such as standardized datasets and analysis code. Other fields such as genomics have demonstrated that resource sharing has the potential to propel research fields extensively beyond state of the art (Kaye et al., 2009; Captur et al., 2016). The benefits will be greater if resource sharing is taken as a new opportunity to answer the many open questions in our fields, rather than a separate independent niche for computational scientists. Taking inspiration from other fields could greatly help us in tackling the potential challenges that come with new opportunities.

## Author Contributions

GD wrote the first draft of the manuscript. JH and NM revised the manuscript. All authors contributed to the article and approved the submitted version.

## Funding

This work was funded by a grant from the National Institutes of Health, NIDCD, DC014279.

## Conflict of Interest

The authors declare that the research was conducted in the absence of any commercial or financial relationships that could be construed as a potential conflict of interest.

## Publisher's Note

All claims expressed in this article are solely those of the authors and do not necessarily represent those of their affiliated organizations, or those of the publisher, the editors and the reviewers. Any product that may be evaluated in this article, or claim that may be made by its manufacturer, is not guaranteed or endorsed by the publisher.
